# Differences in Cancer Care Expenditures and Utilization for Surgery by Hospital Type Among Patients With Private Insurance

**DOI:** 10.1001/jamanetworkopen.2021.19764

**Published:** 2021-08-03

**Authors:** Samuel U. Takvorian, Laura Yasaitis, Manqing Liu, Daniel J. Lee, Rachel M. Werner, Justin E. Bekelman

**Affiliations:** 1 Division of Hematology and Oncology, Perelman School of Medicine, University of Pennsylvania, Philadelphia; 2 Penn Center for Cancer Care Innovation, Abramson Cancer Center, University of Pennsylvania, Philadelphia; 3 Leonard Davis Institute of Health Economics, University of Pennsylvania, Philadelphia; 4 Division of General Internal Medicine, Perelman School of Medicine, University of Pennsylvania, Philadelphia; 5 Division of Urology, Perelman School of Medicine, University of Pennsylvania, Philadelphia; 6 Center for Health Equity Research and Promotion, Corporal Michael J. Crescenz VA Medical Center, Philadelphia, Pennsylvania; 7 Departments of Radiation Oncology and Medical Ethics and Health Policy, Perelman School of Medicine, University of Pennsylvania, Philadelphia

## Abstract

**Importance:**

With rising expenditures on cancer care outpacing other sectors of the US health system, national attention has focused on insurer spending, particularly for patients with private insurance, for whom price transparency has historically been lacking. The type of hospital at which cancer care is delivered may be an important factor associated with insurer spending for patients with private insurance.

**Objective:**

To examine differences in spending and utilization for patients with private insurance undergoing common cancer surgery at National Cancer Institute (NCI) centers vs community hospitals.

**Design, Setting, and Participants:**

This retrospective cross-sectional study included adult patients with an incident diagnosis of breast, colon, or lung cancer who underwent cancer-directed surgery from 2011 to 2014. Mean risk-adjusted spending and utilization outcomes were examined for each hospital type using multilevel generalized linear mixed-effects models, adjusting for patient, hospital, and region characteristics. Data were collected from the Health Care Cost Institute’s national multipayer commercial claims data set, which encompasses claims paid by 3 of the 5 largest commercial health insurers in the United States (ie, Aetna, Humana, and UnitedHealthcare). Data analyses were conducted from February 2018 to February 2019.

**Exposures:**

Hospital type at which cancer surgery was performed: NCI, non-NCI academic, or community.

**Main Outcomes and Measures:**

Spending outcomes were surgery-specific insurer prices paid and 90-day postdischarge payments. Utilization outcomes were length of stay (LOS), emergency department (ED) use, and hospital readmission within 90 days of discharge.

**Results:**

The study included 66 878 patients (51 569 [77.1%] women; 31 585 [47.2%] aged ≥65 years) with incident breast (35 788 [53.5%]), colon (21 378 [32.0%]), or lung (9712 [14.5%]) cancer undergoing cancer surgery at 2995 hospitals (5522 [8.3%] at NCI centers; 10 917 [16.3%] at non-NCI academic hospitals; 50 439 [75.4%] at community hospitals). Treatment at NCI centers was associated with higher surgery-specific insurer prices paid compared with community hospitals ($18 526 [95% CI, $16 650-$20 403] vs $14 772 [95% CI, $14 339-$15 204]; difference, $3755 [95% CI, $1661-$5849]; *P* < .001) and 90-day postdischarge payments ($47 035 [95% CI, $43 289-$50 781] vs $41 291 [95% CI, $40 350-$42 231]; difference, $5744 [95% CI, $1659-9829]; *P* = .006). There were no significant differences in LOS, ED use, or hospital readmission within 90 days of discharge.

**Conclusions and Relevance:**

In this cross-sectional study, surgery at NCI centers vs community hospitals was associated with higher insurer spending for a surgical episode without differences in care utilization among patients with private insurance undergoing cancer surgery. A better understanding of the factors associated with prices and spending at NCI cancer centers is needed.

## Key Points

**Question:**

Are there differences in insurer spending and care utilization for patients with private insurance undergoing cancer surgery at National Cancer Institute (NCI) centers vs community hospitals?

**Findings:**

In this cross-sectional study of 66 878 patients with breast, colon, or lung cancer, surgery at NCI centers, compared with community hospitals, was associated with higher insurer prices paid and higher 90-day postdischarge payments, without differences in length of stay, emergency department use, or hospital readmission.

**Meaning:**

In this study of patients with private insurance undergoing cancer surgery, insurer spending for a surgical episode was higher at NCI centers than community hospitals, without differences in care utilization.

## Teaser

This cross-sectional study examines differences in cancer care spending and utilization for patients with private insurance undergoing common cancer surgical procedures at National Cancer Institute (NCI) centers vs community hospitals.

## Introduction

With rising expenditures on cancer care outpacing other sectors of the US health system,^1,2,3^ national attention has focused on identifying and promoting high-value cancer hospitals, ie, those that consistently deliver excellent outcomes at relatively low cost.^4^ National Cancer Institute (NCI)–designated cancer centers (hereafter NCI centers) are academic hospitals recognized for their scientific and research leadership, training and education programs, and clinical expertise in cancer care.^5^ Treatment at NCI centers may be associated with improved outcomes, particularly for patients with more severe illness and/or more advanced cancers.^6,7,8,9,10^ However, little is known about the degree to which treatment at NCI centers may be associated with higher prices paid or spending for a care episode, limiting an assessment of value in cancer care. Because of their comprehensive service offerings, market share, and prestige, NCI centers may exercise greater leverage in negotiations with commercial insurers, resulting in higher reimbursement rates for cancer care services. This is particularly true for patients with private insurance, for whom health care prices are negotiated between insurers and clinicians and price transparency is lacking.^11^

Therefore, we examined the association between hospital type (NCI center vs community hospital) and insurer spending and care utilization during a surgical episode for patients with private insurance who underwent common cancer surgery. We hypothesized that treatment at NCI centers, compared with community hospitals, would be associated with higher surgery-specific prices paid and episode spending and decreased acute care utilization.

## Methods

### Study Design

We conducted a retrospective cross-sectional study to evaluate the association between hospital type and insurer spending and care utilization during a surgical episode. The study adhered to Strengthening the Reporting of Observational Studies in Epidemiology (STROBE) reporting guidelines^12^ and was exempted by the University of Pennsylvania institutional review board given the use of deidentified data only.

### Data Sources

We used data from the Health Care Cost Institute (HCCI) national multipayer commercial claims data set, which includes claims paid by 3 of the 5 largest commercial health insurers in the United States (ie, Aetna, Humana, and UnitedHealthcare).^13^ The HCCI offers a comprehensive picture of private health insurer spending, encompassing data on more than 40 million covered individuals annually, spanning all 50 states and the District of Columbia. The data set includes reporting of actual prices paid for claims submitted, thus illuminating negotiated transaction prices between clinicians, health systems, and payers.

Hospital-level data were obtained from the 2014 American Hospital Association Annual Survey.^14^ Regional data were captured at the level of the Hospital Referral Region (HRR), encompassing health care markets for tertiary medical care, using publicly available data from the Dartmouth Atlas Project^15^ and 2014 American Community Survey.^16^

### Participants

The study sample included adult patients (age >18 years) with incident breast, colon, or lung cancer who underwent cancer-directed surgery from 2011 to 2014. We selected these cancers because they are common and routinely treated with extirpative surgery in the nonmetastatic setting across both community and academic sites. We used validated, claims-based algorithms, modified for use in private rather than Medicare claims, to identify incident cancer cases and cancer-directed surgical procedures meeting our specifications. To identify patients with incident breast cancer, we applied the multistep validated algorithm described by Nattinger et al.^17^ To identify patients with incident colon or lung cancer, we applied the multistep validated algorithms described by Lavery et al.^18^ Then, we identified cancer-directed surgical procedures by the presence of an *International Classification of Diseases, Ninth Revision* (*ICD-9*) or Healthcare Common Procedure Coding System (HCPCS) procedure code and corresponding primary diagnosis code on an inpatient or outpatient claim (eFigure 1 in the Supplement). If multiple cancer-directed surgical procedures were performed during the study period for a given patient, the first was considered the index surgery. We excluded patients with length of stay in the top 1% (n = 258), as these patients were judged to be outliers who likely had surgical or other complications.

Patient-level data were merged with hospital-level data via an encrypted hospital National Provider Identifier (NPI) present on the claim associated with the index surgery. Thus, patients were attributed to the facility at which their index surgery was performed. Patients with breast cancer undergoing outpatient surgical procedures were attributed to the hospital affiliated with the outpatient place of service; all surgical procedures for colon and lung cancer were performed in the inpatient setting. Patients with missing or nonmerging NPIs were excluded (n = 16 669). HRR-level data were merged based on the zip code of the hospital in which the index surgery was performed. Finally, we limited our analysis to those patients with continuous enrollment for 6 months before and 1 month after the index surgery for baseline covariate and outcome ascertainment, respectively (eFigure 2 in the Supplement).

### Outcomes and Measures

The primary spending outcomes were surgery-specific insurer price paid and 90-day postdischarge payments. For inpatient procedures, the price paid was defined as the sum of all prices paid on surgery-specific facility and physician claims from admission through discharge. For outpatient procedures, the price paid was defined as the sum of all surgery-specific facility and physician claims on the day of the index procedure. Postdischarge spending was calculated by aggregating payments on inpatient, outpatient, physician, and pharmacy claims during the 90-day period after discharge (or date of index surgery for those patients undergoing outpatient procedures). All dollar amounts were adjusted for differences in economic conditions across regions using the Centers for Medicare & Medicaid Services (CMS) Wage Index and were inflated to 2014 dollars (study termination) using the Consumer Price Index for Medical Care.

The primary utilization outcomes were hospital length of stay (LOS), emergency department (ED) use, and hospital readmission within 90 days of discharge. We chose 90-day event rates for our main analysis because CMS considers this interval policy relevant,^19^ and prior studies have advocated for its use.^19,20,21,22,23^

The independent variable was the hospital type at which cancer surgery was performed, separated into 3 mutually exclusive categories: NCI center, non-NCI academic hospital, or community hospital. We used publicly available data to identify hospitals affiliated with an NCI center.^5^ Academic hospitals were identified as those with membership in the Council of Teaching Hospitals and Health Systems of the Association of American Medical Colleges.^24^ Hospitals were attributed to the group corresponding to their highest designation only, and all designations were current as of the year 2014.

Patient-level covariates included age, sex, severity of illness using Elixhauser^25,26^ comorbidities, median household income of the zip code of residence, tumor type, surgical procedure, year of procedure, and enrollment in a Medicare Advantage plan (vs not). We additionally generated a variable of historical monthly spending by aggregating insurer claims paid in the 6 months prior to index surgery and calculating a mean monthly spend over this period.

Hospital-level variables included number of hospital beds, number of intensive care unit beds, annual surgical volume, hospital ownership (ie, for profit, nonprofit, or government), and oncology and chemotherapy services arrangement (ie, hospital owned or joint venture). HRR-level variables included density of physicians, surgeons, specialists, and acute care hospitals. To adjust for regional health care market competition, we calculated the Herfindahl-Hirschman Index by HRR, defined by the share of hospital beds squared, summed over all hospitals in the market area, with higher values indicating less competition.

### Statistical Analysis

We estimated mean adjusted spending and utilization outcomes by hospital type using multilevel mixed-effects generalized linear models, adjusting for patient, hospital, and region characteristics and accounting for clustering of patients within hospitals. Patient-level spending outcomes were modeled with a generalized linear model with log link function and γ distribution, given their positive right-skewed distribution. Patient-level LOS was similarly modeled. Patient-level ED use and hospital readmission were modeled as binary variables using multivariable mixed-effects logistic regression models. After modeling patient-level outcomes, we then estimated mean adjusted spending and utilization outcomes for each hospital type using postestimation predictive margins. The marginal effect of each hospital type was calculated to determine whether the differences in estimated outcomes for NCI centers (and, secondarily, non-NCI academic hospitals) compared with community hospitals were statistically different than zero.

We conducted a sensitivity analysis to test the robustness of our findings to an alternate outcome specification by modeling 30-day event rates for spending and utilization outcomes. All statistical analyses used 2-tailed testing with a significance threshold of *P* < .01 (Bonferroni correction for testing 5 hypotheses across spending and utilization outcomes). Analyses were conducted using SAS version 9.4 (SAS Institute) and Stata version 15.0 (StataCorp).

## Results

### Patient and Hospital Characteristics

We identified 66 878 patients (51 569 [77.1%] women; 31 585 [47.2%] aged ≥65 years) with incident breast (35 788 [53.5%]), colon (21 378 [32.0%]), or lung (9712 [14.5%]) cancer undergoing index cancer surgery at 2995 hospitals (5522 [8.3%] at NCI centers, 10 917 [16.3%] at non-NCI academic hospitals, and 50 439 [75.4%] at community hospitals). Patients treated at NCI centers vs those treated at community hospitals were younger (aged 18-54 years: 2175 [39.4%] vs 12 879 [25.5%]; standardized difference, 0.37) and more likely to be women (4455 [80.7%] vs 38 527 [76.4%]; standardized difference, 0.11). Patients treated at NCI centers had higher historical mean (SD) monthly spending than those treated at community hospitals ($4052 [$6705] vs $2124 [$4292]; standardized difference, 0.34) but similar Elixhauser comorbidity scores (≥3: 1931 [35.0%] vs 17 157 [34.0%]; standardized difference, 0.05). Medicare Advantage coverage was less frequent among those undergoing surgery at NCI centers compared with community hospitals (964 [17.5%] vs 16 270 [32.3%]; standardized difference, 0.35).

NCI centers, compared with community hospitals, were larger (≥200 beds: 59 [96.8%] vs 1031 [37.9%]; standardized difference, 2.60), with higher median (interquartile range) annual surgical volume (30 765 [19 701-47 675] cases vs 5235 [2909-8968] cases; standardized difference, 1.61) and were located in more populated and medically resourced referral regions (median [interquartile range] physicians per 100 000 residents, 223.4 [201.3-259.6] vs 194.8 [180.4-217.3]; standardized difference, 0.90). Lumpectomy was infrequently performed across hospital types (348 of 3248 breast operations [10.7%] at NCI centers vs 2401 of 26 366 [9.1%] at community hospitals). NCI centers, compared with community hospitals, had higher rates of laparoscopic partial colectomy (510 of 1046 colon operations [48.8%] vs 7585 of 17 522 [43.3%]) and pneumonectomy (96 of 1228 lung operations [7.8%] vs 7585 of 17 522 [43.3%]). Table 1 reports other key differences in patient, hospital, and region characteristics by hospital type. The eTable in the Supplement reports characteristics of the study sample as a whole and of those excluded for missing or nonmerging NPI. These groups were similar with standardized differences across measured variables less than 0.1.

**Table 1.** Patient, Hospital, and Region Characteristics, by Hospital Type

Characteristic	No. (%)			Standardized difference, NCI vs community
	NCI	Academic	Community	
Patient characteristics				
Patients, No.	5522 (8.3)	10 917 (16.3)	50 439 (75.4)	
Age, y				
18-44	792 (14.3)	1133 (10.4)	3837 (7.6)	0.37
45-54	1383 (25.1)	2406 (22.0)	9042 (17.9)	
55-64	1477 (26.8)	2963 (27.1)	12 227 (24.2)	
≥65	1868 (33.8)	4413 (40.4)	25 304 (50.2)	
Gender				
Male	1066 (19.3)	2330 (21.3)	11 907 (23.6)	0.11
Female	4455 (80.7)	8587 (78.7)	38 527 (76.4)	
Comorbidities, Elixhauser score				
0	215 (3.9)	423 (3.9)	1692 (3.4)	0.05
1	1554 (28.1)	2978 (27.3)	13 921 (27.6)	
2	1822 (33.0)	3681 (33.7)	17 669 (35.0)	
≥3	1931 (35.0)	3835 (35.1)	17 157 (34.0)	
Income, median (IQR) $^a^	66 403 (47 662-88 638)	62 169 (46 890-82 285)	54 801 (43 163-72 579)	0.40
Medicare Advantage				
Yes	964 (17.5)	2547 (23.3)	16 270 (32.3)	0.35
No	4558 (82.5)	8370 (76.7)	34 169 (67.7)	
Primary tumor				
Breast	3248 (58.8)	6174 (56.6)	26 366 (52.3)	0.39
Colon	1046 (18.9)	2810 (25.7)	17 522 (34.7)	
Lung	1228 (22.2)	1933 (17.7)	6551 (13.0)	
Procedure				
Breast				0.41
Lumpectomy or partial mastectomy	348 (10.7)	624 (10.1)	2401 (9.1)	
Mastectomy	2900 (89.3)	5550 (89.9)	23 965 (90.9)	
Colon				
Partial colectomy, laparoscopic	510 (48.8)	1408 (50.1)	7585 (43.3)	
Partial colectomy, open	490 (46.9)	1325 (47.2)	9673 (55.2)	
Total colectomy	46 (4.4)	77 (2.7)	264 (1.5)	
Lung				
Partial lobectomy	147 (12.0)	195 (10.1)	668 (10.2)	
Lobectomy	985 (80.2)	1605 (83.0)	5499 (83.9)	
Pneumonectomy	96 (7.8)	133 (6.9)	384 (5.9)	
Historical monthly spend, mean (SD), $^b^	4052 (6705)	2927 (4972)	2124 (4292)	0.34
Hospital characteristics				
Hospitals, No.	61 (2.0)	210 (7.0)	2724 (91.0)	
Hospital size				
Small, ≤99 beds	1 (1.6)	3 (1.4)	887 (32.6)	2.60
Medium, 100-199	1 (1.6)	11 (5.2)	806 (29.6)	
Large, 200-500 beds	9 (14.8)	84 (40.0)	890 (32.7)	
Very large, >500 beds	50 (82.0)	112 (53.3)	141 (5.2)	
Intensive care unit size				
None	0	4 (1.9)	282 (10.4)	1.69
Small, 1-5 beds	0	2 (1.0)	266 (9.8)	
Medium, 6-15 beds	3 (4.9)	21 (10.0)	911 (33.4)	
Large, 16-30 beds	7 (11.5)	55 (26.2)	633 (23.2)	
Very large, >30 beds	51 (83.6)	128 (61.0)	632 (23.2)	
Surgical volume, median (IQR)	30 765 (19 701-47 675)	18 688 (11 321-23 957)	5235 (2909-8968)	1.61
Hospital ownership				
Nonprofit	42 (68.9)	158 (75.2)	1801 (66.1)	0.81
For profit	0	7 (3.3)	557 (20.5)	
Government	19 (31.2)	45 (21.4)	366 (13.4)	
Region				
Northeast	17 (28.3)	63 (30.0)	366 (13.5)	0.29
Midwest	12 (20.0)	58 (27.6)	734 (27.1)	
South	20 (33.3)	76 (36.2)	1130 (41.7)	
West	11 (18.3)	13 (6.2)	480 (17.7)	
Region characteristics, median (IQR)				
Total population	528 662 (303 587-868 162)	371 551 (220 035-805 972)	315 548 (151 087-623 611)	0.43
Physician density per 100 000	223.4 (201.3-259.6)	211.5 (190.8-239.7)	194.8 (180.4-217.3)	0.90
Specialist density per 100 000	143.7 (127.6-170.8)	131.6 (120.4-156.3)	123.6 (112.9-136.6)	0.95
Surgeon density per 100 000	40.2 (38.4-46.6)	40.3 (37.5-45.4)	39.2 (37.0-42.1)	0.34
Acute care hospital beds per 1000	2.1 (1.8-2.2)	2.1 (1.8-2.3)	2.1 (1.8-2.4)	0.19

Abbreviations: IQR, interquartile range; NCI, National Cancer Institute.

^a^ Median household income of zip code of patient residence according to 2014 US Census American Community Survey.

^b^ Historical monthly spending calculated as monthly mean spending during 6-month period prior to index surgery.

### Spending Outcomes

Treatment at NCI centers was associated with higher surgery-specific insurer prices paid compared with community hospitals ($18 526 [95% CI, $16 650 to $20 403] vs $14 772 [95% CI, $14 339 to $15 204]; difference, $3755 [95% CI, $1661 to $5849]; *P* < .001), driven predominantly by differences in facility payments ($17 704 [95% CI, $15 845 to $19 563] vs $14 120 [95% CI, $13 691 to $14 549]; difference, $3584 [95% CI, $1525 to $5643]; *P* < .001). We also found that 90-day postdischarge payments were higher at NCI centers compared with community hospitals ($47 035 [95% CI, $43 289 to $50 781] vs $41 291 [95% CI, $40 350 to $42 231]; difference, $5744 [95% CI, $1659 to $9829]; *P* = .006). Table 2 additionally shows spending outcomes at non-NCI academic hospitals, which, compared with community hospitals, had numerically higher surgery-specific insurer prices paid ($16 131 [95% CI, $15 201 to $17 060] vs $14 772 [95% CI, $14 339 to $15 204]; difference, $1359 [95% CI, $280 to $2438]; *P* = .01) and 90-day postdischarge payments ($42 775 [95% CI, $40 824 to $44 726] vs $41 291 [95% CI, $40 350 to $42 231]; difference, $1484 [95% CI, −$775 to $3743]; *P* = .20), but the differences were not statistically significant.

**Table 2.** Differences in Adjusted Insurer Spending by Hospital Type^a^

Spending category	Adjusted price and total spending, $			NCI center vs community hospital		Academic hospital vs community hospital	
	NCI center, mean (95% CI)	Academic hospital, mean (95% CI)	Community hospital, mean (95% CI)	Difference (95% CI), $	*P* value	Difference (95% CI), $	*P* value
**Surgery-specific price**							
Facility	17 704 (15 845 to 19 563)	15 394 (14 469 to 16 319)	14 120 (13 691 to 14 549)	3584 (1525 to 5643)	<.001	1274 (204 to 2344)	.02
Physician	1912 (1769 to 2054)	1731 (1657 to 1805)	1667 (1631 to 1704)	244 (5.5 to 483)	.05	64 (−34 to 162)	.20
Total	18 526 (16 650 to 20 403)	16 131 (15 201 to 17 060)	14 772 (14 339 to 15 204)	3755 (1661 to 5849)	<.001	1359 (280 to 2438)	.01
**30-d Postdischarge spending**							
Inpatient	24 586 (21 941 to 27 231)	21 375 (20 077 to 22 672)	20 254 (19 635 to 20 872)	4332 (803 to 7862)	.02	1121 (−463 to 2705)	.17
Outpatient	5213 (4532 to 5893)	5075 (4681 to 5470)	5482 (5275 to 5688)	−269 (−73 417 to 72 879)	.99	−406 (−11 0842 to 11 0029)	.99
Physician	7996 (7221 to 8770)	7826 (7395 to 8257)	7806 (7569 to 8044)	189 (−675 to 1053)	.67	20 (−485 to 525)	.94
Pharmacy	366 (307 to 425)	332 (301 to 363)	298 (284 to 311)	69 (−7825 to 7962)	.99	35 (−3941 to 4010)	.99
Total	32 692 (29 785 to 35 599)	29 782 (28 278 to 31 285)	28 390 (27 662 to 29 119)	4301 (1102 to 7500)	.008	1392 (−349 to 3132)	.12
**90-d Postdischarge spending**							
Inpatient	26 514 (23 662 to 29 366)	22 923 (21 530 to 24 315)	21 644 (20 984 to 22 304)	4870 (−1054 to 10793)	.11	1278 (−800 to 3357)	.23
Outpatient	14 449 (12 380 to 16 517)	12 992 (11 909 to 14 076)	11 670 (11 189 to 12 152)	2778 (550 to 5007)	.02	1322 (99 to 2545)	.03
Physician	12 095 (10 850 to 13 339)	12 344 (11 622 to 13 066)	13 331 (12 909 to 13 754)	−1236 (−2644 to 172)	.09	−987 (−1850 to −124)	.03
Pharmacy	1180 (986 to 1374)	1023 (924 to 1121)	967 (920 to 1013)	213 (4 to 423)	.05	56 (−57 to 170)	.33
Total	47 035 (43 289 to 50 781)	42 775 (40 824 to 44 726)	41 291 (40 350 to 42 231)	5744 (1659 to 9829)	.006	1484 (−775 to 3743)	.20

Abbreviation: NCI, National Cancer Institute.

^a^ Patient-level surgery-specific price and total episode spending were modeled using a generalized linear model with log link function and γ distribution, adjusting for patient, hospital, and region characteristics and calculating robust standard errors accounting for clustering of patients within hospitals. After modeling patient-level outcomes, the marginal effect of each hospital type was calculated using postestimation predictive margins to determine whether the differences in estimated outcomes for NCI and academic hospitals vs community hospitals were statistically significant.

### Utilization Outcomes

There were no significant differences by hospital type in LOS, ED utilization, or hospital readmission within 90 days. Mean LOS was comparable at NCI centers and community hospitals (5.1 [95% CI, 4.8-5.4] days vs 5.1 [95% CI, 5.1-5.2] days, *P* = .73). The probability of ED utilization (13.1% [95% CI, 11.9%-14.3%] vs 13.2% [95% CI, 12.8%-13.5%]; *P* = .93) or hospital readmission (10.4% [95% CI, 9.2%-11.5%] vs 10.8% [95% CI, 10.5%-11.1%]; *P* = .48) within 90 days was also similar between NCI centers and community hospitals. Table 3 additionally highlights utilization outcomes at non-NCI academic hospitals compared with community hospitals which were not significantly different.

**Table 3.** Differences in Adjusted Utilization by Hospital Type^a^

Utilization category	Adjusted utilization			NCI center vs community hospital		Academic hospital vs community hospital	
	NCI center, mean (95% CI)	Academic hospital, mean ([95% CI)	Community hospital, mean (95% CI)	Difference (95% CI)	*P* value	Difference (95% CI)	*P* value
Length of stay, d	5.1 (4.8 to 5.4)	5.0 (4.9 to 5.2)	5.1 (5.1 to 5.2)	−0.1 (−0.4 to 0.2)	.73	−0.1 (−0.3 to 0.1)	.22
Emergency department visit, %							
30-d Postdischarge	8.1 (7.2 to 9.1)	8.1 (7.5 to 8.7)	7.6 (7.3 to 7.9)	0.5 (−0.5 to 1.6)	.33	0.5 (−0.2 to 1.2)	.15
90-d Postdischarge	13.1 (11.9 to 14.3)	13.2 (12.4 to 14.0)	13.2 (12.8 to 13.5)	−0.1 (−1.4 to 1.3)	.93	0 (−0.9 to 0.9)	.97
Hospital readmission, %							
30-d Postdischarge	6.4 (5.5 to 7.3)	6.5 (5.9 to 7.0)	6.7 (6.5 to 7.0)	−0.3 (−1.3 to 0.6)	.49	−0.3 (−0.9 to 0.4)	.43
90-d Postdischarge	10.4 (9.2 to 11.5)	10.5 (9.8 to 11.2)	10.8 (10.5 to 11.1)	−0.5 (−1.7 to 0.8)	.48	−0.3 (−1.2 to 0.5)	.47

Abbreviation: NCI, National Cancer Institute.

^a^ Patient-level length of stay was modeled using a generalized linear model with log link function and γ distribution. Patient-level emergency department visit and hospital readmission were modeled as binary variables using multivariable mixed-effects logistic regression models. All models were adjusted for patient, hospital, and region characteristics and used robust standard errors accounting for clustering of patients within hospitals. After modeling patient-level outcomes, the marginal effect of each hospital type was calculated using postestimation predictive margins to determine whether the differences in estimated outcomes for NCI centers and academic hospitals vs community hospitals were statistically significant.

### Sensitivity Analyses

The results of sensitivity analyses modeling 30-day event rates across spending and utilization outcomes are included in Table 2 and Table 3. These results were consistent with those of our main analyses, with patients treated at NCI centers incurring higher 30-day post-discharge payments than those treated at community hospitals ($32 692 [95% CI, $29 785-$35 599] vs $28 390 [95% CI, $27 662-$29 119]; difference, $4301 [95% CI, $1102-$7500]; *P* = .008), without significant differences in rates of ED utilization (8.1% [95% CI, 7.2%-9.1%] vs 7.6% [95% CI, 7.3%-7.9%]; *P* = .33) or hospital readmission (6.4% [95% CI, 5.5%-7.3%] vs 6.7% [95% CI, 6.5%-7.0%]; *P* = .49).

## Discussion

Using a national multipayer commercial claims data set, we found that surgery-specific and 90-day postdischarge spending were higher at NCI centers than community hospitals and in an intermediate range at non-NCI academic hospitals without differences in acute care utilization for patients with private insurance undergoing surgery for breast, colon, or lung cancer. Facility rather than physician payments accounted for most of the differences in spending outcomes, consistent with national trends showing that hospital payments occupy a disproportionate and growing share of overall health care spending.^27^ These results support our hypothesis that insurer spending would be higher at NCI centers than community hospitals, possibly due to their size, market share, and prestige, affording leverage in negotiations with private payers. However, contrary to our hypothesis, there were comparable rates of postdischarge acute care utilization across hospital types, suggesting that negotiated transaction prices rather than utilization may be driving site-level differences in spending.

To our knowledge, this is the first study to report on variations in insurer prices paid and episode spending by hospital type for privately insured patients undergoing common cancer surgery. Prior research has explored spending variation for older patients with cancer covered by Medicare.^28,29,30,31,32,33,34^ However, because prices are administered rather than negotiated under Medicare, these data shed little insight into insurer spending patterns for patients with private insurance. Whereas utilization is the primary driver of spending for Medicare beneficiaries, price is an additional driver of spending for those with private insurance.^35,36^ Consistent with this, we found meaningful differences by hospital type in both surgery-specific insurer prices paid and 90-day postdischarge payments without concomitant differences in postdischarge acute care utilization.

With national trends of health care consolidation,^37^ health systems increasingly encompass both community and academic sites and are faced with strategic decisions about how to rationalize care services across facilities. Many have advocated for a hub and spoke model, with regionalization of complex care at academic referral centers, such as NCI centers, and more routine care delivered at community sites.^38,39,40,41,42^ This stems in part from evidence suggesting improved outcomes for patients with cancer treated at NCI centers^6,7,8,9,10^ and academic hospitals,^43,44,45,46,47^ particularly for those with more advanced and complex disease. This study focused on common cancer surgical procedures and illuminates important price differences that should factor into such decisions, revealing that there is a premium associated with receipt of surgical cancer care at NCI centers. While acceptable to pay higher prices for care that is expected to be of higher quality, we found no differences in short-term postsurgical outcomes (90-day ED utilization and hospital readmission) by hospital type, which is consistent with results from a recent study comparing postsurgical outcomes for Medicare patients across varying types of cancer hospitals.^48^ Further research examining hospital-level differences in long-term postsurgical outcomes, such as mortality, paired with spending outcomes, is necessary to judge whether and under what circumstances the premium price of NCI centers is justified.

This study has other important implications. For commercial payers, who are keenly aware that discrepancies in hospital negotiated prices contribute to premium growth, our findings suggest an incentive to steer patients away from high-cost hospitals. Consistent with this, research has shown that insurers are increasingly excluding oncologists affiliated with NCI centers from narrow health care networks.^49^ For health systems operating in a predominantly fee-for-service environment, our findings suggest an incentive to maximize surgical volume at more lucrative referral centers, despite upward pressure on total health care spending. Value-based or bundled payment reimbursement for surgical episodes, particularly when paired with mandatory reporting on surgical outcomes, could help to rectify this misalignment.

Finally, for policy makers who have long touted the prospects of price transparency to help consumers comparison shop across institutions and prepare for expected financial burden, our findings underscore the continued importance of such efforts. A historical lack of transparency into negotiated transaction prices for individuals with private insurance has led to limited available data on the prices paid for hospital services. Beginning January 1, 2019, CMS mandated that all hospitals publish their chargemasters, which detail standard prices for all hospital services and procedures. However, these list prices bear little resemblance to what is actually charged or ultimately paid by patients and payers.^50,51^ Moreover, there is substantial variability in the availability, accessibility, and comprehensiveness of published chargemasters, further degrading the effectiveness of this policy.^52^ A more recent executive order by former President Trump mandates disclosure of negotiated prices between insurers, hospitals, and physicians. Future research will be necessary to evaluate the effects of this policy, which took effect on January 1, 2021.^53^

### Limitations

This study has limitations. First, the analysis was limited to patients with private insurance undergoing cancer-directed surgery for breast, colon, or lung cancer, and may not generalize to patients with other malignant neoplasms or those receiving nonsurgical cancer care. However, these are 3 of the 4 most common incident cancers and make up most of cancer surgical volumes nationally, suggesting that our results may at least extend to other oncologic surgical populations. Second, this study did not analyze patient out-of-pocket spending and thus cannot infer the degree to which patients were exposed to the observed price and spending differences. Future research should explore this important dimension of spending. Third, while every attempt was made to adjust for differences in case mix, our claims-based analysis did not allow for complete adjustment of clinical factors such as stage at diagnosis, surgical complexity, and pathologic status, which limited our ability to judge surgical quality. However, we used validated algorithms to identify incident cancer diagnoses and cancer-directed surgical procedures, which are likely to identify, predominantly, patients with early-stage cancer fit enough for cancer surgery. Also, we paired spending outcomes with utilization outcomes, which provide at least high-level insight into hospital quality of care. Additionally, due to the observational nature of this study, observed differences by hospital type may be attributable to unmeasured factors, including differences in coding intensity or clinical severity, unmeasured in our data sets.

## Conclusions

In this study of patients with private insurance undergoing surgery for incident breast, colon, or lung cancer, surgery at NCI centers, compared with community hospitals, was associated with higher insurer spending across a surgical care episode without differences in care utilization. A better understanding of the drivers of prices and spending at NCI centers is needed.

## Notes

Supplement. **eFigure 1. **Procedure Codes Used to Identify Cancer-Directed Surgical Procedures **eFigure 2. **Study Flow Diagram **eTable.** Patient Characteristics of Study Population and of Those Excluded Due to Missing or Nonmerging National Provider Identifiers
