# Supplementary material for: Differences in Cancer Care Expenditures and Utilization for Surgery by Hospital Type Among Patients With Private Insurance
Source: JAMA Netw Open. 2021 Aug 3;4(8):e2119764. doi: 10.1001/jamanetworkopen.2021.19764 (PMC8335573; doi:10.1001/jamanetworkopen.2021.19764)
Supplement: Supplement. — eFigure 1. Procedure Codes Used to Identify Cancer-Directed Surgical Procedures eFigure 2. Study Flow Diagram eTable. Patient Characteristics of Study Population and of Those Excluded Due to Missing or Nonmerging National Provider Identifiers [file jamanetwopen-e2119764-s001.pdf]

Supplemental Online Content

Takvorian SU, Yasaitis L, Liu M, Lee DJ, Werner RM, Bekelman JE. Differences in cancer care expenditures and utilization for surgery by hospital type among patients with private insurance. *JAMA Netw Open.* 2021;4(8):e2119764. doi:10.1001/jamanetworkopen.2021.19764

eFigure 1. Procedure Codes Used to Identify Cancer-Directed Surgical Procedures

eFigure 2. Study Flow Diagram

eTable. Patient Characteristics of Study Population and of Those Excluded Due to Missing or Nonmerging National Provider Identifiers

This supplemental material has been provided by the authors to give readers additional information about their work.

© 2021 Takvorian SU et al. *JAMA Network Open.*

eFigure 1. Procedure Codes Used to Identify Cancer-Directed Surgical Procedures

COHORT OF INCIDENT BREAST CANCER CASES

- Lumpectomy/Partial mastectomy
  - ICD9-CM: 85.20-85.21; 85.22-85.23
  - CPT/HCPCS: 19120, 19125, 19126; 19160, 19162; 19301, 19302
- Mastectomy
  - ICD9-CM: 85.33-85.48 (including 85.4)
  - CPT/HCPCS: 19180-19255; 19303-19307

COHORT OF INCIDENT COLON CANCER CASES

- Partial colectomy (laparoscopic)
  - ICD9-CM: 17.3, 17.31-17.36, 17.39
  - CPT/HCPCS: 44204-44208, 44210, 44212
- Partial colectomy (open)
  - ICD9-CM: 45.7, 45.71-45.76, 45.79
  - CPT/HCPCS: 44140, 44141, 44143-44147, 44160
- Total colectomy
  - ICD9-CM: 45.8, 45.81-45.83

COHORT OF INCIDENT LUNG CANCER CASES

- Partial lobectomy
  - ICD9-CM: 32.3, 32.30, 32.39
- Lobectomy
  - ICD9-CM 32.4, 32.41, 32.49
- Pneumonectomy
  - ICD9-CM 32.5, 32.50, 32.59, 32.6

© 2021 Takvorian SU et al. *JAMA Network Open.*

eFigure 2. Study Flow Diagram

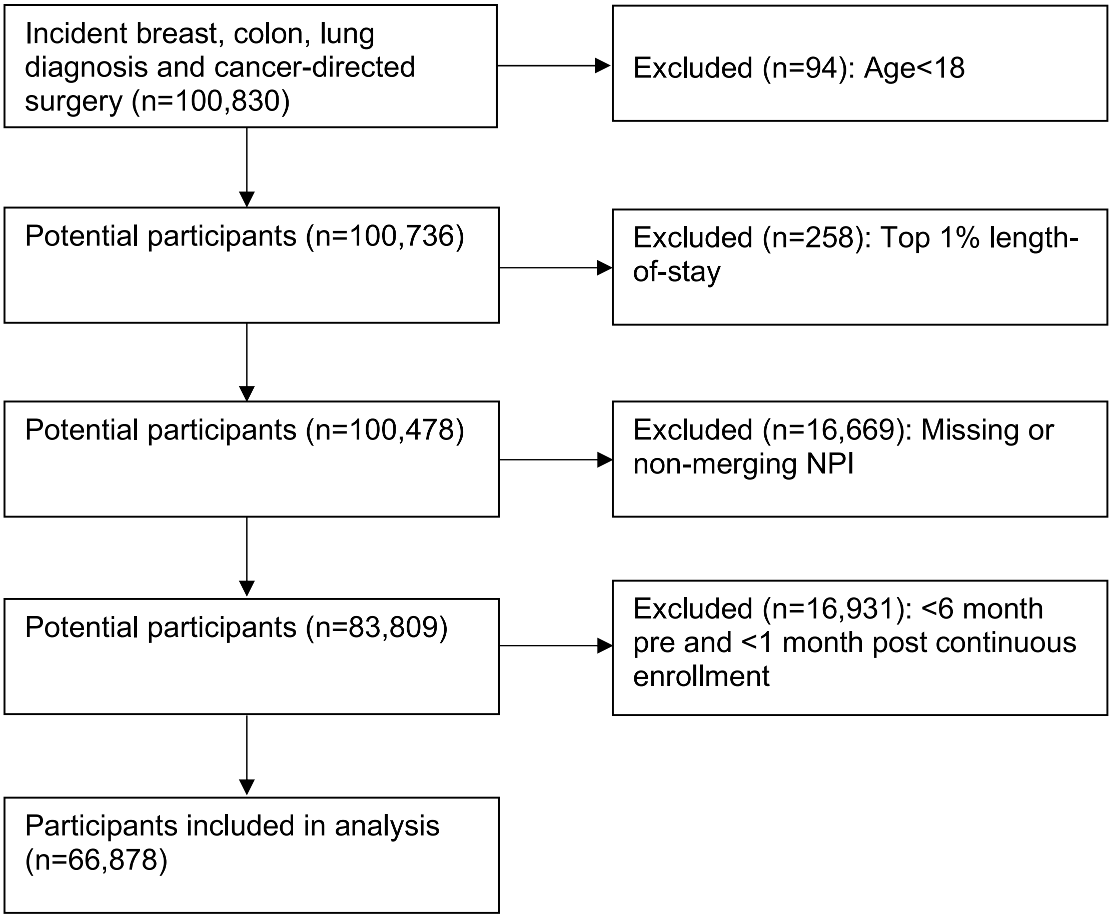

© 2021 Takvorian SU et al. *JAMA Network Open.*

eTable. Patient Characteristics of Study Population and of Those Excluded Due to Missing or Nonmerging National Provider Identifiers (NPI)

Characteristic	Study population<br>N = 66,878	Missing/Non-merging NPI<br>N = 16,669	Standardized difference
Patient characteristics			
Age, n (%)			0.045
18-44	5762 (8.6)	1570 (9.4)	
45-54	12831 (19.2)	3099 (18.6)	
55-64	16667 (24.9)	3966 (23.8)	
65+	31585 (47.2)	8016 (48.1)	
Gender, n (%)			0.008
Male	15303 (22.9)	3759 (22.6)	
Female	51569 (77.1)	12908 (77.4)	
Comorbidities [Elixhauser], n (%)			0.055
0	2330 (3.5)	462 (2.8)	
1	18453 (27.6)	4592 (27.6)	
2	23172 (34.6)	5586 (33.5)	
3+	22923 (34.3)	6029 (36.2)	
Median Income, $ [q1, q3] *	56616 [43897, 75513]	55506 [43541, 74407]	0.035
Medicare Advantage, n (%)			0.084
Yes	19781 (29.6)	5584 (33.5)	
No	47097 (70.4)	11085 (66.5)	
Primary tumor, n (%)			0.034
Breast	35788 (53.5)	9149 (54.9)	
Colon	21378 (32.0)	5069 (30.4)	
Lung	9712 (14.5)	2451 (14.7)	
Procedure, n (%)			0.076
Breast			
Lumpectomy/Partial mastectomy	3373 (9.4)	1117 (12.2)	
Mastectomy	32415 (90.6)	8032 (87.8)	
Colon			
Partial colectomy (laparoscopic)	9503 (44.5)	2285 (45.1)	
Partial colectomy (open)	11488 (53.7)	2697 (53.2)	
Total colectomy	387 (1.8)	87 (1.7)	
Lung			
Partial lobectomy	1010 (10.4)	278 (11.3)	
Lobectomy	8089 (83.3)	2007 (81.9)	
Pneumonectomy	613 (6.3)	166 (6.8)	
Historical monthly spending, $ mean (SD) **	2414 (4687)	2911 (5558)	0.097
NPI= National Provider Identifier			
NCI= National Cancer Institute			
* Median household income of ZIP of patient residence according to 2014 U.S. Census American Community Survey			
** Historical monthly spending calculated as monthly average spending over 6-month period prior to index surgery			

© 2021 Takvorian SU et al. *JAMA Network Open.*
